# Percutaneous coronary intervention as an independent predictor of non-target lesion progression in 1658 patients with coronary artery disease

**DOI:** 10.7150/thno.125363

**Published:** 2026-01-14

**Authors:** Xiaoling Liu, Lin Chen, Chaoyu Liu, Zeyuan Mei, Jiaqi Li, Yuan Zhang, Meiling Chang, Haowei Zhang, Chenghu Guo, Mei Zhang, Guipeng An, Jianmin Yang, Wenqiang Chen, Yuzeng Xue, Cheng Zhang, Mei Ni, Yun Zhang

**Affiliations:** 1 State Key Laboratory for Innovation and Transformation of Luobing Theory; Key Laboratory of Cardiovascular Remodeling and Function Research of MOE, NHC, CAMS and Shandong Province; Department of Cardiology, Qilu Hospital of Shandong University, Jinan, China.; 2 Clinical Epidemiology Unit, Qilu Hospital of Shandong University, Jinan, China.; 3 Cardiovascular Disease Research Center of Shandong First Medical University, Central Hospital Affiliated to Shandong First Medical University, Jinan, China.; 4 Department of Cardiology, Liaocheng People's Hospital, Liaocheng, China.; 5 Department of Cardiology, Affiliated Hospital of Shandong Second Medical University, Weifang, China.

**Keywords:** non-target lesions, coronary artery disease, stenosis index, percutaneous coronary intervention, fasting blood glucose

## Abstract

**Rationale:** Non-target lesions (NTLs) progression is common in patients with coronary artery disease (CAD). However, its predictors remain obscure.

**Methods:** An angiographic study was conducted in patients with CAD who underwent coronary angiography twice at an interval of 6 to 30 months. NTLs were defined as lesions not treated with percutaneous coronary intervention (PCI) during the first hospitalization. A stenosis index (SI) was calculated from all NTLs in each patient. NTLs progression was defined as an increase in SI (ΔSI > 0) at follow-up.

**Results:** Among 1658 patients recruited, 1061 (64.0%) exhibited NTL progression, with a ΔSI of 0.75 (0.40, 1.30) over a mean follow-up period of 13 months. The NTLs progression group had more males, diabetics, higher neutrophil ratio, creatinine, fasting blood glucose (FBG), uric acid, more PCI therapy and higher SI on the first admission, and higher systolic blood pressure, heart rate, serum levels of low-density lipoprotein cholesterol and FBG at readmission. Multiple logistic regression analysis identified male sex, PCI therapy, and SI on the first admission, and FBG on the second admission were independent predictors of NTLs progression, with the odds ratio of 1.390 (95%CI 1.034~1.869), 1.375 (95%CI 1.087~1.740), 1.003 (95%CI 1.002~1.004) and 1.184 (95% CI 1.086~1.291), respectively.

**Conclusions:** Over 60% of CAD patients developed NTL progression within 30 months. Male sex, PCI therapy and SI on the first admission, and FBG on the second admission were independent predictors of NTLs progression.

## Introduction

The impact of non-target lesions (NTLs) on recurrent major cardiovascular adverse events (MACE) has been a long-standing clinical concern. The PROSPECT study reported that the cumulative recurrence rate of MACE in patients with acute coronary syndrome (ACS) was 20.4% within 3 years after percutaneous coronary intervention (PCI) 1. Of these, 12.9% and 11.6% were related to the original culprit and non-culprit lesions, respectively 1. The PROSPECT II study further reported that 13.2% of patients with myocardial infarction relapsed within 4 years of the first attack, 8% of which was caused by initial non-culprit lesions 2. Seo *et al.* found that about 20% of patients receiving PCI underwent revascularization at the site of coronary artery lesions that were initially not significantly stenotic (≤ 70% stenosis) and not intervened during a mean follow-up of 4.2 years 3.

Traditional atherosclerotic risk factors such as hyperglycemia and dyslipidemia play an important role in the progression of NTLs. Wang J *et al.* reported that 42.7% of the patients had NTLs progression 12 months after PCI and fasting blood glucose (FBG) was positively correlated with the progression of NTLs 4. In a study by Sekimoto T *et al.*, 20.4% of patients with ACS and undergoing primary PCI showed rapid progression of NTLs at the 10-month follow-up coronary angiography, which was significantly associated with small dense low-density lipoprotein cholesterol levels 5. However, clinical observations indicated that many patients with well-controlled risk factors and single-vessel disease still developed NTLs soon after PCI, suggesting that inherent atherosclerosis risk factors may not be sufficient to explain the occurrence of NTLs after PCI.

In 1998, Gaspardone *et al*. found that the serum level of C-reactive protein (CRP) was significantly increased after PCI in patients with stable coronary artery disease (CAD), which predicted higher cardiovascular events during follow-up 6. This finding was confirmed by subsequent studies, which further revealed that in patients treated with high-dose statins, NTLs still showed a tendency for accelerated progression during 2-year follow-up after PCI, suggesting a lipid-independent mechanism 7,8. In addition, recent clinical studies demonstrated that elevated serum levels of inflammatory cytokines independently predicted adverse cardiovascular events even after use of second-generation drug-eluting stents 9. Similarly, Xu *et al.* followed up patients with stable CAD treated with PCI and found that plasma CRP independently predicted rapid progression of NTLs 10. Recently, we performed an experiment in a rabbit model of atherosclerosis, and found that stent implantation triggered acute phase response and systemic inflammation, which was associated with increased plaque burden and pathological features of unstable plaque in NTLs 11. More recently, Zuo *et al.* measured the perivascular fat attenuation index (FAI), an imaging marker of coronary inflammation, in patients who underwent pre- and post-stenting coronary computed tomography angiography, and found that both perivascular FAI and plaque volume in NTLs increased significantly after stenting, indicating a focal inflammation progression after coronary stenting 12. These studies suggested a possible relation between stent implantation and systemic inflammation.

Taken together, previous studies focused on traditional atherosclerotic risk factors as a major cause of progression of NTLs, while neglecting the effects of current treatment of CAD including PCI and pharmacological therapy. In order to comprehensively assess all possible factors affecting the progression of NTLs, we conducted an angiographic study in patients with CAD who were admitted for coronary angiography twice at a time interval of 6 to 30 months at Qilu Hospital of Shandong University in the past 10 years and analyzed the clinical and imaging data in these patients to find independent predictors of NTLs progression.

## Methods

### Study design and population

**Figure 1:**
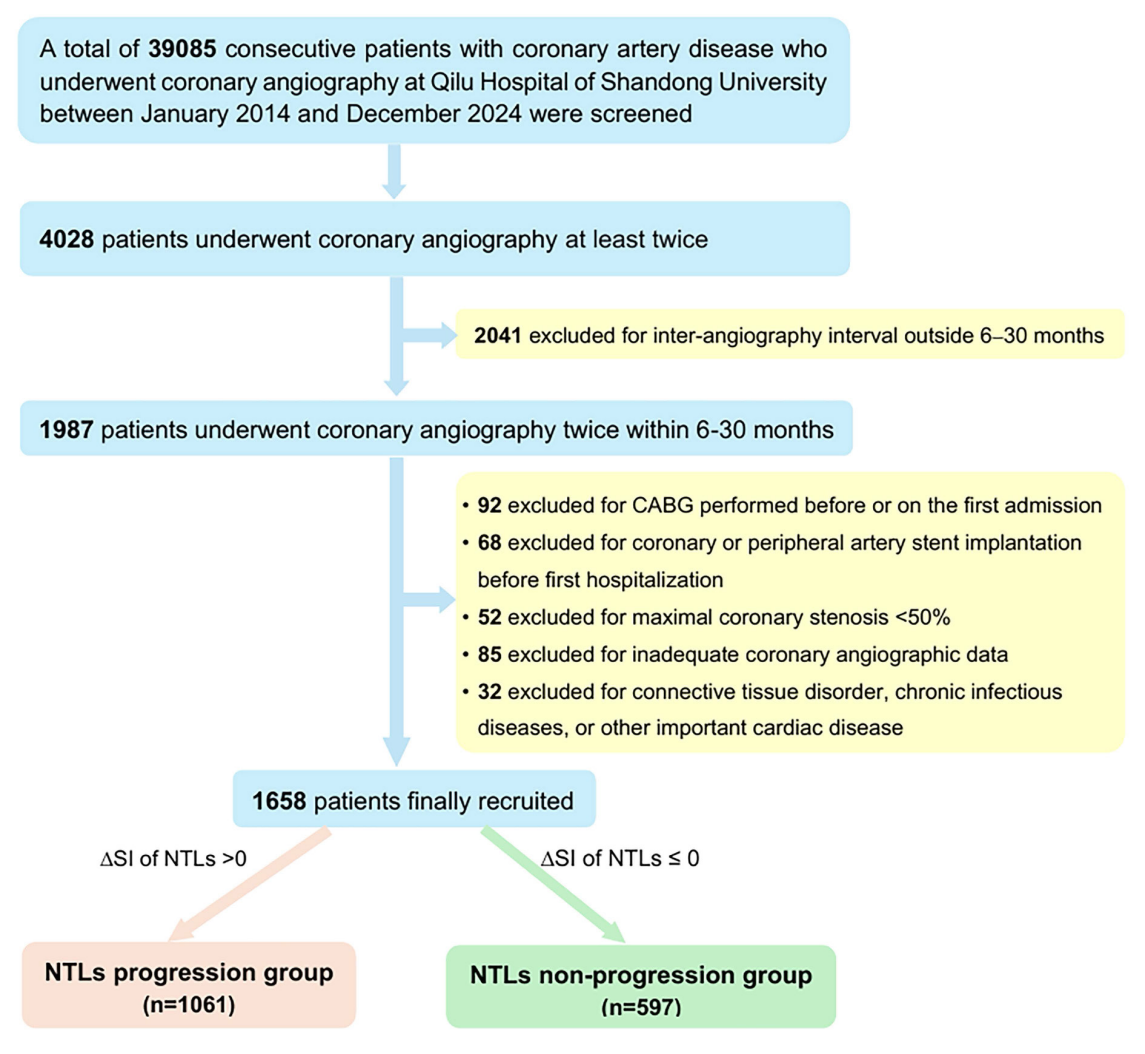
** Flow chart of patient screening.** CABG, coronary artery bypass surgery; CAD, coronary artery disease; NTLs, non-target lesions.

Clinical records of patients admitted for myocardial ischemic symptoms at Qilu Hospital of Shandong University in the last 10 years (from January 2014 to December 2024) were reviewed for potential enrollers of the study (Figure 1). The inclusion criteria were: (1) at least 18 years of age; (2) coronary artery diameter stenosis was ≥ 50% on first admission; and (3) coronary angiography was performed twice at a time interval of 6 to 30 months. This interval was chosen to ensure detectable angiographic changes while minimizing confounding effects due to natural progression of atherosclerosis 7,8. The exclusion criteria were: (1) coronary artery bypass grafting (CABG) was performed before or during the first hospitalization; (2) coronary angiographic images were technically inadequate for quantitative analysis; (3) clinically important diseases other than CAD including connective tissue disorders, chronic infectious diseases, aortic stenosis, congenital heart disease, intracardiac thrombosis, and coronary dissection. The study was approved by the Ethics Committee on Scientific Research of Qilu Hospital of Shandong University [No. 2021(217)].

### Data collection

The medical database of Qilu Hospital of Shandong University from January 2014 to December 2024 was searched, and all data of patients who were admitted during this time period and met the inclusion criteria but did not meet the exclusion criteria listed above were collected, including age, sex, medical history, physical examination records, laboratory assays, coronary angiographic images, PCI images, and drug administration. The changes (Δ) for the laboratory variable were calculated using the formula: Δ = (value on the second admission) - (value on the first admission).

### Quantitative coronary angiography

Two experienced cardiac interventionists independently evaluated all imaging data included in coronary angiography and/or interventional therapy. The videos of coronary angiography recorded twice in the same patient were evaluated at the same angle. The lumen diameters of the stenotic segment of NTLs and the adjacent normal segment (reference segment) were measured *de novo* using Radiant DICOM Viewer (64-bit) software (Medixant, Poland). The lumen diameters of the narrow and normal segment were compared and luminal stenosis was calculated using the following formula: lesion diameter stenosis rate (%) = [1 - stenotic segment diameter/reference segment diameter] × 100%. The whole burden of coronary artery stenosis of NTLs for each patient was quantitated by stenosis index (SI) using the formula: 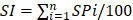, where SP = stenosis percentage of each NTL.

To evaluate intra-observer and inter-observer variability in SI measurement, a total of 30 angiograms were randomly selected and the SI of NTLs were measured. Intra-observer variability was assessed by the same expert who measured SI of NTLs twice four-weeks apart. Interobserver variability was assessed by two experts who measured SI of NTLs in a blinded way. Bland-Altman plots were used to analyze the inter-observer and intra-observer variability and intraclass correlation coefficients (ICC) were calculated.

### Definition of the progression of non-target lesions (NTLs)

In the present study, NTLs were defined as all coronary artery lesions which were not treated with PCI during first hospitalization. If NTLs were located in the same artery as the PCI-treated lesion, the criterion of a ≥ 10 mm distance from either stent edge was applied to exclude stent-edge effects, in accordance with the methodology used in prior studies 13, 14.

**Figure 2:**
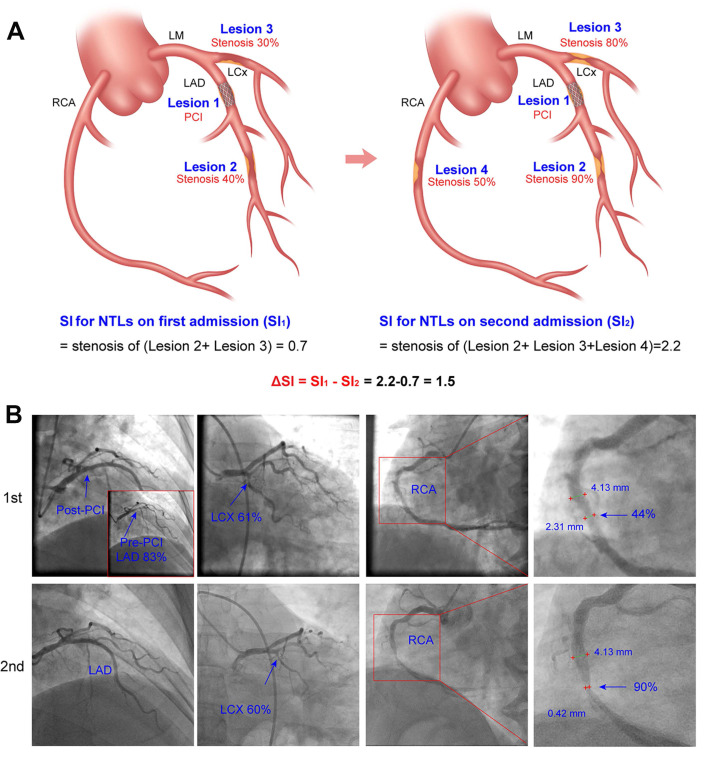
** Schematic diagram and representative images of quantitative coronary angiography showing coronary artery stenosis and progression of non-target lesions. A.** Schematic diagram for calculating coronary artery stenosis index (SI) and progression of NTLs. Left, Lesion 1 had been treated with PCI. SI_1_ for NTLs on the first admission = stenosis of Lesion 2 (0.40) + stenosis of Lesion 3 (0.30) = 0.70. Right, SI_2_ for NTLs on the second admission = stenosis of Lesion 2 (0.90) + stenosis of Lesion 3 (0.80) + stenosis of Lesion 4 (0.50) = 2.2. ΔSI for NTLs= SI_2_ - SI_1_ = 2.2 - 0.7 = 1.5. **B.** Representative images of quantitative coronary angiography. Sequential coronary angiography (CAG) in a 57-year-old male with unstable angina pectoris on the first admission and with acute myocardial infarction on the second admission. Upper row: First CAG revealed diffuse lesions in the proximal LAD with 83% stenosis, a lesion with 61% stenosis in the distal LCX, and a lesion with 44% stenosis in the mid RCA. A rapamycin-eluting stent was implanted in the lesion of LAD. SI_1_ for NTLs = 0.61 + 0.44 = 1.05; Lower row (6-month later): Second CAG showed 90% stenosis of the mid-RCA (previously 44% stenosis). SI_2_ for NTLs = 0.90 + 0.60 = 1.50. After an interval of 6 months, progression of the NCLs was quantitated by ΔSI =1.50 - 1.05 = 0.45. LAD, left anterior descending artery; CAG, coronary angiography, LCX, left circumflex artery; RCA, right coronary artery.

NTLs progression was assessed using the following formula: ΔSI = SI_2_ - SI_1_, where SI_2_ and SI_1_ were SI values of NTLs in each patient on the second and the first admission, respectively, and patients with ΔSI ≤ 0 and ΔSI > 0 were defined as NTLs non-progression group and NTLs progression group, respectively. Figure 2 showed the method for measuring SI and calculating ΔSI in NTLs.

### Primary endpoint and patient grouping

The primary endpoint of this study was the progression of NTLs within 6 to 30 months after the first coronary angiography. Patients were divided into two groups based on ΔSI for NTLs: NTLs progression group with ΔSI > 0 and NTLs non-progression group with ΔSI ≤ 0. The clinical, biochemical and coronary angiographic characteristics of patients in both groups were analyzed and compared.

### Statistical analysis

Data were analyzed using SPSS Version 27.0 for Windows (IBM, Armonk, New York) and R Version 4.4.1 (R Foundation for Statistical Computing). All data were tested for normality and homogeneity of variance. Continuous data that followed a normal distribution were expressed as mean ± standard deviation (SD), and comparisons between two groups were performed using the independent samples t-test. Otherwise, data were presented as median with interquartile range [M (Q1, Q3)], and the Mann-Whitney U test was employed for group comparisons. Categorical variables were expressed as percentages and analyzed using the Chi-square test. The inter-observer and intra-observer agreement for SI of NTLs were analyzed by ICC. Univariate logistic regression was used to analyze the association between clinical and imaging values, and NTLs progression. Multiple logistic regression analysis was performed to identify independent predictors of NTLs progression. The selection of variables in the multivariate model was based on a combination of statistical and clinical criteria. All variables that showed a significant association with the outcome in univariate analyses (*P* < 0.05) were retained. In addition, conventional atherosclerotic risk factors (age, sex, history of hypertension and diabetes, smoking status, LDL-C level, and blood pressure) were incorporated into the model based on their established clinical relevance as documented in the literature. The goodness-of-fit of the model was evaluated using the Hosmer-Lemeshow test. Multicollinearity among the variables was evaluated using variance inflation factors (VIF). Subgroup analysis was conducted to assess the consistency of the predictive effect of PCI therapy on NTLs progression among 12 prespecified subgroups. *P <* 0.05 was considered statistically significant.

## Results

### Study population

The flow chart diagram of patient screening was presented in Figure 1. A total of 39085 consecutive patients with CAD who underwent coronary angiography at Qilu Hospital of Shandong University between January 2014 and December 2024 were initially screened. Among them, 4028 patients underwent coronary angiography at least twice but only 1987 patients underwent coronary angiography twice within a predefined time interval of 6-30 months. Ultimately, 1658 patients (1203 men, aged 60.44 ± 9.84 years) who met the inclusion criteria and none of the exclusion criteria were included in the final analysis. A comparison between the final analytical cohort (n = 1658) and eligible-but-not-included patients (n = 2370) showed no significant differences in their baseline characteristics, supporting the representativeness of the study cohort for the entire CAD patient population who underwent coronary angiography at Shandong University Qilu Hospital during the last 10 years (Table S1).

During the first hospitalization, the diagnosis was acute myocardial infarction in 432 cases, unstable angina pectoris in 1048 cases, stable angina pectoris in 150 cases and previous myocardial infarction in 28 cases. All patients underwent coronary angiography and 1236 patients (74.6%) underwent PCI with drug-eluting stent implantation during the first hospitalization. Based on ΔSI of NTLs, 1061 (64%) patients (794 men, 60.48 ± 9.99 years) belonged to NTLs progression group, while 597 (36%) patients (408 men, 60.37 ± 9.58 years) belonged to the NTLs non-progression group (Figure 1).

### Baseline characteristics of patients at the first admission

There were more male patients (74.8% vs. 68.3%, *P =* 0.004) and more patients with diabetes (33.4% vs. 27.0%, *P =* 0.007) in the NTLs progression group than in the NTLs non-progression group (Table 1). Patients in the NTLs progression group showed higher proportion of neutrophils, and serum levels of creatinine, fasting blood glucose (FBG) and uric acid than those in the NTLs non-progression group (all *P <* 0.05, Table 1). There was no significant difference in the age, history of hypertension, history of smoking, blood pressure and heart rate between the two groups.

Baseline coronary angiography during the first hospitalization showed that there was no significant difference in the proportion of patients with severe coronary stenosis (coronary lumen diameter stenosis ≥ 75%) between the two groups (*P =* 0.574). There were 30.2% of patients in the NTLs-progression group and 32.3% in non-NTLs-progression group with severe three-vessel disease, respectively (*P =* 0.359). During the first admission, a total of 818 patients underwent PCI therapy in the NTLs progression group, while only 418 patients received PCI therapy in the NTLs non-progression group (77.1% vs. 70.0%, *P =* 0.001). The SI in the NTLs progression group was higher than that in the NTLs non-progression group in the first hospitalization [1.70 (0.90, 2.65) vs. 1.20 (0.50, 2.30), *P <* 0.001] (Table 1).

### Clinical characteristics of patients on the second admission

The time interval between the first and the second admission and coronary angiography was 13 (8, 20) months in the finally recruited population, in whom this time interval was 13 (8, 21) months in the NTLs progression group and 12 (8, 18.5) months in the NTLs non-progression group, with no significant difference between the two groups. More patients experienced acute myocardial infarction in the NTLs progression group than those in the NTLs non-progression group (13.57% vs. 7.87%, *P <* 0.05). Systolic blood pressure and heart rate were higher in the NTLs progression group than in the NTLs non-progression group on the second admission (both *P <* 0.05). There was no significant difference in diastolic blood pressure and smoking cessation rates after the first angiography between the two groups (Table 2).

Serum levels of low-density lipoprotein cholesterol (LDL-C) and FBG were significantly higher in the NTLs progression group compared to the NTLs non-progression group (all *P* < 0.05). No significant differences were observed in the serum concentrations of other biochemical parameters between the two groups (Table 2). The changes in these laboratory measurements between the first and second admissions did not differ significantly between the NTLs progression and NTLs non-progression groups (all *P* > 0.05; Table S2).

### NTLs progression on the second angiography

In the NTLs progression group, the ΔSI for NTLs relative to the first coronary angiography was 0.77 (0.40, 1.40). Among these, progression occurred only in NTLs located in the same coronary artery as the original target lesions (termed homologous NTLs) in 149 patients, with a ΔSI of 0.50 (0.40, 0.80); Progression occurred only in NTLs situated in different coronary arteries from the original target lesions (termed non-homologous NTLs) in 670 patients, with a ΔSI of 0.60 (0.40, 1.10); Progression involving both non-homologous and homologous NTLs was observed in 242 patients, with a ΔSI for NTLs of 1.40 (0.90, 2.00). Overall, homologous NTLs progression occurred in 391 patients with a ΔSI of 0.50 (0.40, 0.80), and non-homologous NTLs progression occurred in 912 patients with a ΔSI of 0.70 (0.40, 1.15).

Consequently, 589 patients (55.5%) in the NTLs progression group underwent coronary revascularization due to myocardial ischemia during the second hospitalization, including 538 patients (50.7%) who underwent PCI and 51 (4.8%) who underwent CABG. In contrast, 104 patients (17.4%) in the NTLs non-progression group required revascularization, including 95 who underwent PCI and 9 who underwent CABG. Chi-square tests revealed statistically significant differences between the two groups in the incidence of MACE, which was defined as a composite of acute myocardial infarction and revascularization on the second admission. Both the incidence of acute myocardial infarction (13.6% vs. 7.9%, *P* < 0.001) and the rate of coronary revascularization (55.5% vs. 17.4%, *P* < 0.001) were significantly higher in the NTLs progression group than in the NTLs non-progression group. As results, the total MACE was substantially elevated in the NTLs progression group compared to the NTLs non-progression group (60.9% vs. 22.9%, *P* < 0.001, Table 2). These findings underscored a strong association between NTLs progression and adverse cardiovascular outcomes.

### Univariate logistic regression

Univariate logistic regression analysis was performed to unveil the potential predictors of NTLs progression. The analysis showed that male sex, history of diabetes, blood neutrophil ratio, serum levels of creatinine and FBG, PCI therapy, and SI of NTLs on the first admission, as well as SBP and serum FBG level on the second admission, were significantly associated with NTLs progression (Table 3).

### Binary logistic regression

To adjust for potential confounders and identify independent binary logistic regression was performed using NTLs progression as the dependent variable. Independent variables included all predictors of NTLs progression in univariate analyses, including neutrophil ratio, serum levels of creatinine and FBG, PCI therapy, and SI of NTLs at the first admission, as well as SBP and serum FBG on the second admission, in addition to conventional atherosclerotic risk factors such as age, male sex, history of diabetes, hypertension, smoking, LDL-C level. The goodness-of-fit of the logistic regression model was confirmed by the Hosmer-Lemeshow test (χ² = 14.584, P = 0.068), showing no evidence of lack of fit. Male sex, PCI therapy, and SI of NTLs on the first admission, and FBG on the second admission were identified as independent predictors of NTLs progression. Among these, male sex (OR = 1.390, 95% CI: 1.034-1.869) and PCI therapy (OR = 1.375, 95% CI: 1.087-1.740) were notable predictors. Additionally, SI of NTLs on the first admission (OR = 1.003, 95% CI: 1.002-1.004) and FBG on the second admission (OR = 1.184, 95% CI: 1.086-1.291) were also independent predictors, both showing high statistical significance (P < 0.001) (Table 4).

### Subgroup analysis of the predictive effect of PCI on NTLs progression

Subgroup analysis was further performed to examine the consistency of the predictive effect of PCI on the progression of NTLs across different patient populations. The predictive effect of PCI on NTLs progression was consistent across all subgroups (all P for interaction > 0.100). No significant interactions were observed between PCI therapy during the first admission and any of the following variables: sex, age, history of diabetes, hypertension, smoking, SBP, FBG, LDL-C levels on both the first and second admissions, and SI during the first admission.

**Figure 3:**
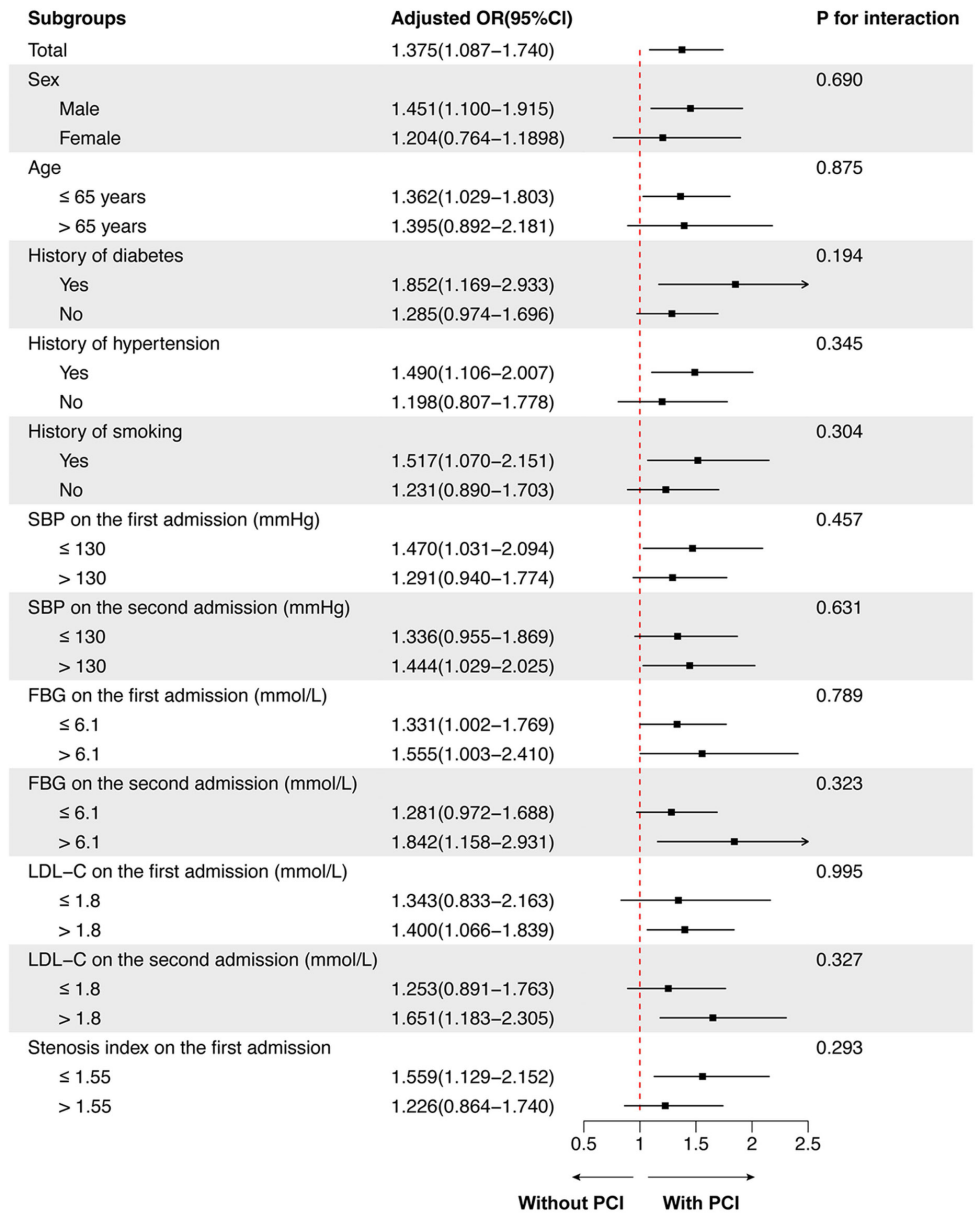
** Subgroup analysis for the predictive effect of PCI on NTLs progression.** The predictive effect of PCI on NTLs progression was analyzed in 12 prespecified subgroups. FBG, serum level of fasting blood glucose; LDL-C, low-density lipoprotein cholesterol; PCI, percutaneous coronary intervention; SBP, systolic blood pressure.

Although the overall effect was consistent, the magnitude of the predictive effect of PCI on NTLs progression varied across subgroups. Specifically, the effect appeared more pronounced in patients with a history of diabetes (OR = 1.852, 95% CI: 1.169-2.933), LDL-C > 1.8 mmol/L on the second admission (OR = 1.651, 95% CI: 1.183-2.305), and FBG > 6.1 mmol/L on the second admission (OR = 1.842, 95% CI: 1.158-2.931). In contrast, the effect was less marked in females (OR = 1.204, 95% CI: 0.764-1.898), patients aged > 65 years (OR = 1.395, 95% CI: 0.892-2.181), and those without a history of hypertension (OR = 1.198, 95% CI: 0.807-1.778). These variations, however, did not reach statistical significance in interaction tests, suggesting that predictive effect of PCI therapy on NTLs progression remained consistent across the studied populations (Figure 3).

### Inter-observer and intra-observer variability of stenosis index measurement

Bland-Altman plots were shown in Figure S1. Excellent reproducibility was observed in the measurement of SI. The intra-observer ICCs for SI were 0.985 and 0.975 for the two observers, respectively, while the inter-observer ICC was 0.983 (Figure S1).

## Discussion

**Figure 4:**
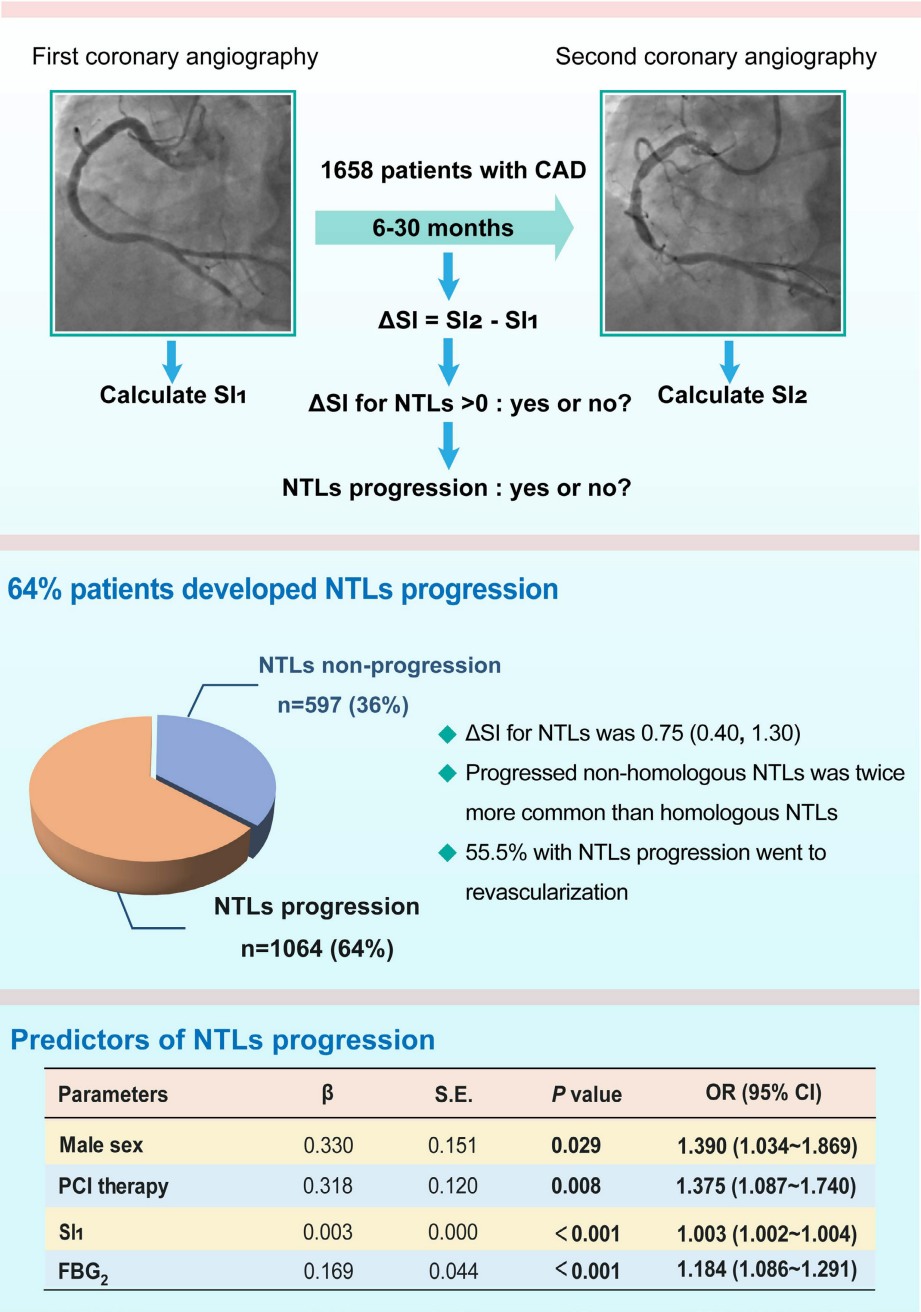
** A schematic illustration showing the main findings of this study.** CAD, coronary artery disease; FBG_2_, fasting blood glucose on the second admission; NTLs, non-culprit lesions; PCI, percutaneous coronary intervention; SI, stenosis index, which was obtained by the formula: 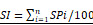, where SP = stenosis percentage of each coronary lesion. SI_1_ and SI_2_, SI values of NTLs in each patient on the first and the second admission, respectively.

There were several important findings in the present study (Figure 4). First, more than 60% patients with CAD developed rapid NTLs progression within 6 to 30 months and the median of ΔSI for NTLs was 0.75 at a mean follow-up of 13 months. Progressed non-homologous NTLs was twice more common than homologous NTLs. Importantly, more than half patients in the NTLs-progression group underwent coronary revascularization due to myocardial ischemia. Second, male sex, history of diabetes, neutrophil ratio, serum level of creatinine, FBG, PCI therapy and SI on the first admission, and SBP and the serum levels of FBG on the second admission were significantly correlated with NTLs progression. Third, male sex, PCI therapy and SI on the first admission, and serum level of FBG on the second admission were independent predictors of NTLs progression. To the best of our knowledge, our study was the first in the literature to report the clinical and angiographic characteristics, therapeutic significance of early detection and PCI per se as a key predictor of progression of NTLs.

The findings of the present study have important clinical implications. First, more than half of the recruited patients developed NTLs progression at a mean follow-up of 13 months, which suggests that the progression speed of NTLs was so high that NTLs may elicit frank myocardial ischemia one year after PCI and thus, a close post-discharge follow-up in these patients is necessary. Second, in patients with previous PCI therapy, progressed non-homologous NTLs was twice more common than homologous NTLs, suggesting that NTLs progression involved multiple coronary arteries, which may not be limited to the intervened vessel. Third, more than half of the patients in the NTLs progression group underwent coronary revascularization due to myocardial ischemia during the second hospitalization, indicating the high severity of these progressed lesions and the importance of early detection. These results suggested that development of a novel therapy to lower the incidence of NTLs progression may ultimately reduce coronary revascularization rate in patients with CAD.

It has been well established that traditional atherosclerotic risk factors may promote the progression of NTLs. Consistent with previous reports, our study showed that patients with NTLs progression often had multiple inherent atherosclerotic risk factors, including male sex, history of diabetes, elevated serum levels of LDL-C, FBG and uric acid, and more severe stenotic lesions 4,5,15,16. Furthermore, we found that SI on the first admission and FBG levels on the second admission were significant predictors of NTLs progression. Similarly, Wang *et al.* reported that FBG was positively correlated with non-culprit lesion progression, with FBG ≥ 5.715 mmol/L as a predictor, in a 12-month follow-up of STEMI patients after primary PCI 4. The predictive role of baseline SI was consistent with and extended previous findings from our group and others. This was strongly supported by the PROSPECT study, which demonstrated that non-culprit lesions associated with recurrent MACE typically had a plaque burden ≥ 70%, a minimal luminal area ≤ 4.0 mm², or thin-cap fibroatheroma features 1. PROSPECT II further identified large lipid-rich plaques and high plaque burden as independent predictors of non-culprit lesion-related MACE 2. In a recent study, Usui *et al.* found that untreated non-culprit lesions exhibiting thin-cap fibroatheroma and minimum lumen area < 3.5 mm², were associated with increased risk of non-culprit-related MACE 14. The strong association between SI and NTLs progression in the current study suggested that the overall atherosclerotic burden reflected pan-coronary vulnerability, indicative of a more aggressive and widespread disease process.

However, our study was different from previous ones because all patients admitted for CAD were recruited no matter whether they received PCI or not, and all clinical factors were taken into account including both traditional atherosclerotic risk factors and interventional and pharmacological treatment. The effect of PCI on the long-term prognosis of patients with stable coronary disease has been highly controversial 17. Recently, Jo SH *et al.* compared the effects PCI plus optimal medical therapy (OMT) versus OMT only on the long-term prognosis of a total of 11346 patients with stable angina in a matched cohort study. They found that the adverse cardiovascular events were significantly higher in the PCI+OMT group than in the OMT group in a mean follow-up of 9.3 years 18. The mechanism underlying this paradoxical finding was unclear but may be related to NTLs progression induced by PCI therapy as demonstrated in the present study. Our results sounded a warning to the growing clinical applications of PCI in patients with stable coronary disease, and called for a need to develop a preventive and therapeutic strategy against PCI-accelerated atherosclerosis. To this end, the exact mechanism propelling NTLs progression after PCI is to be clarified. Our previous study suggested that chronic inflammation after stent implantation may play an important role 11. In an experiment in a rabbit model of atherosclerosis, we implanted a drug-eluting stent at the target lesions of the abdominal aorta, and found that NTLs of rabbits with stent implantation showed greater plaque load and vulnerability than those of rabbits without stent implantation, suggesting that stent implantation may promote the progression of NTLs 11. To disclose the potential mechanism, serum proteomic analysis was performed, which revealed that the expression of 7 proteins was upregulated remarkably in rabbits with stent implantation, of which 6 proteins belonged to acute phase proteins (APPs). These results indicated that APPs and APPs-triggered chronic inflammation may be the key mechanism of stenting-related NTLs progression.

The increased expression of serum inflammatory factors after PCI has attracted attention from cardiologists for many years 19. Despite these advances in stent technology, inflammation remains a concern in patients with CAD after PCI 9. In our recent prospective study involving 147 patients with CAD, we found there was a surge in the serum levels of serum amyloid A-1 (SAA-1), CRP, tumor necrotic factor-α (TNF-α), and interleukin (IL)-6 one day after PCI, and the serum levels of CRP, TNF-α, and IL-6 remained high even one month after the procedure. In contrast, patients undergoing coronary angiography alone only exhibited a slight increase in the serum levels of CRP, SAA-1, and IL-6 one day after the procedure, which returned to baseline levels one month later 11. It has been reported that the acute inflammation induced by PCI was likely triggered by direct endothelial damage, liberation of intra-plaque proinflammatory debris, and reperfusion injury 20. However, the mechanism behind the persisting chronic inflammation after PCI is different from that of acute inflammation early after PCI, and the specific mechanism remains elusive. Two mechanisms might be responsible. First, although stent-eluted drugs, such as rapamycin and everolimus, may effectively inhibit smooth muscle cell proliferation and occurrence of restenosis after stent implanation, these drugs may delay coronary artery endothelization and allow continuous release of inflammatory cytokines from the damaged vascular wall induced by stenting. Second, stent implantation results in loss of contractile function of the stenting segment, with a low shear stress inside the segment 21, which may increase endothelial permeability, expression of monocyte chemotactic protein-1 (MCP-1) and macrophage infiltration as shown by our previous study 22. Regardless of which mechanism involved, increased chronic inflammation after PCI with stent implantation may promote rapid NTLs progression, which may lead to coronary revascularization, yielding to a vicious cycle of “PCI begets PCI”, as revealed by this study. These findings raised a possibility that preoperative anti-inflammation therapy may inhibit post-PCI inflammation and NTLs progression, ideally leading to an improved long-term outcome in patients undergoing PCI 9,23.

### Study limitations

This study contains several limitations. First, this study was conducted in a single medical center, which may induce patient selection bias. However, all patients admitted to our department during the last 10 years who met the inclusion criteria but did not meet the exclusion criteria were recruited to minimize selection bias and all coronary angiographic images were prospectively analyzed with a quantitative software. Univariate and binary logistic regression were performed to rule out the effects of interactive factors and subgroup analyses were undertaken to examine the consistency of the predictive effect of PCI on the progression of NTLs across different patient populations. Second, our study was limited by the lack of systematic data of medication adherence and lifestyle interventions. The progression of atherosclerotic lesions is highly influenced by systemic medical therapy and risk factor control. Therefore, the causal relationship between PCI and NTLs progression can only be established in a multicenter clinical trial with CAD patients randomized to PCI or medical treatment to observe the long-term difference in NTLs progression and MACE. Third, although a quantitative software was used to measure the angiographic coronary artery dimeter with a high precision up to 10 μm and a strict criterion was applied to define NCLs progression as ΔSI > 0, plaque morphology and stability were not assessed using sophisticated techniques such as intravascular ultrasound or optical coherence tomography. Finally, systematic inflammatory biomarkers (e.g., CRP, IL-6) were not routinely measured in our CAD cohort, which limited our ability to examine the association between NTLs progression and systemic inflammation in the current study. Further clinical studies are warranted to overcome all these limitations.

## Conclusions

More than 60% patients with CAD developed NTLs progression within 6 to 30 months, and more than half of these patients underwent coronary revascularization due to myocardial ischemia. Male sex, history of diabetes, neutrophil ratio, serum level of creatinine, FBG, PCI therapy and SI on the first admission, and SBP and the serum levels of FBG on the second admission were significantly correlated with NTLs progression. Male sex, PCI therapy, a high SI on the first admission, and elevated FBG on the second admission were identified as independent predictors of the progression of NTLs. Development of new therapies targeting PCI-evoked inflammation may reduce the incidence of NTLs progression and cardiovascular events after PCI. A randomized clinical trial is needed to confirm these preliminary findings.

## Supplementary Material

Supplementary tables and figure S1.

## Figures and Tables

**Table 1:** Baseline characteristics of patients in NTLs progression and non-progression groups on the first admission

Parameters	NTLs progression group (n = 1061)	NTLs non-progression group (n = 597)	*P* value
Age (years)	60.48±9.99	60.37±9.58	0.839
Sex (male, %)	794 (74.8%)	408 (68.3%)	0.004
SBP (mmHg)	133 (120, 146)	132 (119, 145)	0.272
DBP (mmHg)	76 (68, 85)	76 (68, 83)	0.449
HR (bpm)	72 (64, 80)	71 (64, 80)	0.380
History of hypertension (n, %)	695 (65.5%)	378 (63.3%)	0.371
History of diabetes (n, %)	354 (33.4%)	161 (27.0%)	0.007
History of smoking (n, %)	538 (50.7%)	282 (47.2%)	0.175
Principal diagnosis			0.188
Acute MI (n, %)	294 (27.7%)	138 (23.1%)	
UAP (n, %)	652 (61.5%)	396 (66.3%)	
SAP (n, %)	98 (9.2%)	52 (8.7%)	
Previous MI (n, %)	17 (1.6%)	11 (1.8%)	
Drug administration			
Aspirin (n, %)	1033 (97.4%)	576 (96.5%)	0.365
P2Y12R inhibitor (n, %)	982 (92.6%)	558 (93.5%)	0.551
ACEI/ARB/ARNI (n, %)	557 (52.5%)	287 (48.1%)	0.084
β-receptor blocker (n, %)	723 (68.1%)	402 (67.3%)	0.736
Statins (n, %)	998 (94.1%)	568 (95.1%)	0.896
CCB (n, %)	346 (32.6%)	178 (29.8%)	0.240
Laboratory assays			
Neutrophil ratio (%)	63.40 (57.90, 69.65)	62.58 (56.75, 67.20)	0.004
TC (mmol/L)	3.88 (3.28, 4.61)	3.88 (3.32, 4.52)	0.687
LDL-C (mmol/L)	2.27 (1.83, 2.87)	2.22 (1.83, 2.81)	0.384
HDL-C (mmol/L)	1.06 (0.92, 1.21)	1.07 (0.93, 1.25)	0.239
TG (mmol/L)	1.38 (1.02, 1.86)	1.35 (1.06, 1.77)	0.812
Creatinine (μmol/L)	72.00 (63.00, 81.50)	71.00 (61.00, 79.00)	0.007
FBG (mmol/L)	5.52 (4.80, 6.88)	5.31 (4.68, 6.27)	< 0.001
Uric acid (μmol/L)	320.00 (278.00, 350.00)	316.00 (253.00, 364.00)	0.032
Homocysteine (μmol/L)	14.80 (11.45, 17.70)	14.50 (11.00, 18.05)	0.501
Coronary angiographic data			
Extent of significantly stenotic coronary lesions (≥75%) (n, %)			0.574
Single-vessel lesions	301 (28.4%)	177 (29.6%)	
Two-vessel lesions	351 (33.1%)	183 (30.7%)	
Three-vessel lesions	320 (30.2%)	193 (32.3%)	
Patients undergoing PCI (n, %)	818 (77.1%)	418 (70.0%)	0.001
Stenosis index of NTLs	1.70 (0.90, 2.65)	1.20 (0.50, 2.30)	< 0.001

The continuous variables were expressed as the median (Q1, Q3). ACEI, angiotensin converting enzyme inhibitor; ARB, Angiotensin receptor blocker; ARNI, angiotensin receptor neprilysin inhibitor; CCB, calcium channel blocker; DBP, diastolic blood pressure; FBG, fasting blood glucose; HbA1C, glycosylated hemoglobin, type A1C; HDL-C, high-density lipoprotein cholesterol; HR, heart rate; bpm, beats per minute; LDL-C, low-density lipoprotein cholesterol; MI, myocardial infarction; NTLs, non-target lesions; SBP, systolic blood pressure; PCI, percutaneous coronary intervention; SAP, stable angina pectoris; TC, total cholesterol; TG, triglycerides; UAP, unstable angina pectoris. Stenosis index was obtained by the formula: 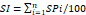, where SP = percentage stenosis of each non-target lesion.

**Table 2:** Characteristics of patients in NTLs progression and NTLs non-progression groups on the second admission

Parameters	NTLs progression group (n = 1061)	NTLs non-progression group (n = 597)	*P* value
Principal diagnosis Acute MI (n, %)	144 (13.6%)	47 (0.8%)	0.001
UAP (n, %)	766 (72.2%)	458 (76.7%)	
SAP (n, %)	98 (9.2%)	71 (11.9%)	
Previous MI (n, %)	53 (5.0%)	21 (3.5%)	
Revascularization (n, %)	589 (55.5%)	104 (17.4%)	< 0.001
MACE (n, %)	646 (60.9%)	137 (22.9%)	< 0.001
SBP (mmHg)	132 (121, 144)	129 (118, 141)	0.009
DBP (mmHg)	74 (68, 83)	74 (67, 82)	0.265
HR (bpm)	70 (63, 78)	68 (62, 77)	0.045
Neutrophil ratio (%)	63.00 (57.40, 68.10)	62.50 (57.20, 67.50)	0.220
TC (mmol/L)	3.30 (2.88, 3.93)	3.30 (2.85, 3.88)	0.426
LDL-C(mmol/L)	1.86 (1.44, 2.27)	1.78 (1.46, 2.15)	0.048
HDL-C(mmol/L)	1.03 (0.91, 1.20)	1.05 (0.92, 1.18)	0.485
TG (mmol/L)	1.24 (0.93, 1.65)	1.26 (0.92, 1.59)	0.593
Creatinine (μmol/L)	72.00 (63.00, 82.00)	72.00 (62.50, 81.00)	0.083
FBG (mmol/L)	5.58 (4.85, 6.61)	5.21 (4.69, 6.05)	< 0.001
Uric acid (μmol/L)	315.00 (275.50, 341.50)	309.00 (251.50, 360.00)	0.149
Homocysteine (μmol/L)	13.70 (10.90, 17.65)	13.50 (10.85, 17.65)	0.799

The continuous variables were expressed as the median (Q1, Q3). MACE was defined as a composite of acute myocardial infarction and revascularization during the second admission in this study. bpm, beats per minute; DBP, diastolic blood pressure; FBG, fasting blood glucose; HbA1C, glycosylated hemoglobin, type A1C; HDL-C, high-density lipoprotein cholesterol; HR, heart rate; LDL-C, low-density lipoprotein cholesterol; MACE, major adverse cardiovascular events; MI, myocardial infarction; NTLs, non-target lesions; SAP, stable angina pectoris; SBP, systolic blood pressure; TC, total cholesterol; TG, triglycerides; UAP, unstable angina pectoris.

**Table 3:** Univariate logistic regression analysis for risk factors of NTLs progression

Variates	β	S.E.	Wald χ2	*P*	OR (95% CI)
Age	0.001	0.005	0.046	0.830	1.001 (0.991~1.011)
Male sex	0.320	0.113	8.049	0.005	1.378 (1.104~1.719)
History of hypertension	0.095	0.107	0.800	0.371	1.100 (0.893~1.356)
History of diabetes	0.305	0.113	7.276	0.007	1.356 (1.087~1.692)
History of smoking	0.139	0.102	1.840	0.175	1.149 (0.940~1.405)
Parameters on first admission					
SBP	0.003	0.003	1.565	0.211	1.003 (0.998~1.009)
DBP	0.003	0.004	0.582	0.445	1.003 (0.995~1.012)
HR	0.002	0.004	0.159	0.690	1.002 (0.993~1.010)
Neutrophil ratio	0.015	0.006	6.838	0.009	1.015 (1.004~1.026)
TC	0.056	0.050	1.231	0.267	1.057 (0.958~1.166)
LDL-C	0.082	0.064	1.651	0.199	1.085 (0.958~1.230)
HDL-C	-0.151	0.204	0.544	0.461	0.860 (0.577~1.283)
TG	0.002	0.048	0.001	0.971	1.002 (0.912~1.101)
Creatinine	0.005	0.002	4.062	0.044	1.005 (1.000~1.009)
FBG	0.074	0.028	6.966	0.008	1.077 (1.019~1.137)
Uric acid	0.001	0.001	0.948	0.330	1.001 (0.999~1.002)
Homocysteine	0.003	0.008	0.206	0.650	1.003 (0.989~1.018)
three-vessel lesions with seriously stenosis	0.101	0.110	0.840	0.359	1.106 (0.891~1.373)
PCI therapy on the first admission	0.366	0.115	10.043	0.002	1.442 (1.150~1.807)
Stenosis index on the first admission	0.003	0.000	34.541	< 0.001	1.003 (1.002~1.003)
Parameters on the second admission					
SBP	0.007	0.003	6.171	0.013	1.007 (1.002~1.013)
DBP	0.004	0.005	0.908	0.341	1.004 (0.995~1.014)
HR	0.007	0.005	2.323	0.128	1.007 (0.998~1.017)
Neutrophil ratio	0.004	0.006	0.534	0.465	1.004 (0.993~1.015)
TC	0.042	0.058	0.522	0.470	1.043 (0.931~1.168)
LDL-C	0.114	0.077	2.205	0.138	1.212 (0.964~1.304)
HDL-C	-0.139	0.205	0.458	0.499	0.870 (0.582~1.301)
TG	0.076	0.066	1.306	0.253	1.079 (0.947~1.229)
Creatinine	0.002	0.001	1.870	0.171	1.002 (0.999~1.004)
FBG	0.182	0.036	24.96	< 0.001	1.200 (1.117~1.288)
Uric acid	0.001	0.001	1.011	0.315	1.001 (0.999~1.002)
Homocysteine (μmol/L)	-0.001	0.007	0.022	0.881	0.999 (0.985~1.013)
Changes in laboratory indices between the two admissions					
ΔNeutrophil ratio	-0.008	0.005	2.786	0.095	0.992 (0.982~1.001)
ΔTC	-0.024	0.049	0.231	0.631	0.977 (0.887~1.076)
ΔLDL-C	-0.003	0.061	0.002	0.966	0.997 (0.885~1.124)
ΔHDL-C	0.017	0.232	0.005	0.942	1.017 (0.645~1.803)
ΔTG	0.056	0.057	0.981	0.322	1.058 (0.947~1.182)
ΔCreatinine	0.000	0.002	0.040	0.841	1.000 (0.994~1.003)
ΔFBG	0.051	0.029	3.172	0.075	1.053 (0.995~1.114)
ΔUric acid	0.000	0.001	0.002	0.965	1.000 (0.999~1.001)
ΔHomocysteine (μmol/L)	-0.007	0.009	0.535	0.464	0.993 (0.976~1.011)

DBP, diastolic blood pressure; FBG, fasting blood glucose; HbA1C, glycosylated hemoglobin, type A1C; HDL-C, high-density lipoprotein cholesterol; HR, heart rate; LDL-C, low-density lipoprotein cholesterol; NTLs, non-target lesions; PCI, percutaneous coronary intervention; SBP, systolic blood pressure; TC, total cholesterol; TG, triglycerides.

**Table 4:** Multivariate logistic regression analysis for predictive factors of NTLs progression.

Parameters	β	S.E.	Wald χ2	*P* value	OR (95% CI)
Male sex	0.330	0.151	4.769	0.029	1.390(1.034~1.869)
Age	-0.002	0.006	0.090	0.765	0.998(0.988~1.009)
History of hypertension	-0.033	0.117	0.081	0.777	0.967(0.769~1.217)
History of diabetes	0.057	0.134	0.182	0.670	1.059(0.815~1.376)
History of smoking	-0.073	0.131	0.311	0.577	0.930(0.720~1.201)
SBP on the first admission	0.002	0.003	0.475	0.491	1.002(0.996~1.008)
Neutrophil ratio on the first admission	0.010	0.006	3.136	0.077	1.010(0.999~1.022)
Creatinine on the first admission	0.004	0.002	2.425	0.119	1.004(0.999~1.008)
FBG on the first admission	-0.002	0.034	0.003	0.957	0.998(0.933~1.068)
LDL-C on the first admission	0.050	0.072	0.487	0.485	1.052(0.913~1.211)
PCI therapy on the first admission	0.318	0.120	7.029	0.008	1.375(1.087~1.740)
Stenosis index on the first admission	0.003	0.000	34.221	<0.001	1.003(1.002~1.004)
SBP on the second admission	0.006	0.003	3.295	0.069	1.006(1.000~1.012)
FBG on the second admission	0.169	0.044	14.719	<0.001	1.184(1.086~1.291)
LDL-C on the second admission	0.068	0.087	0.613	0.434	1.070(0.903~1.269)

FBG, serum level of fasting blood glucose; LDL-C, low-density lipoprotein cholesterol; NTLs, non-target lesions; PCI, percutaneous coronary intervention; SBP, systolic blood pressure.

## Abbreviations

ACS: acute coronary syndrome
APPs: acute phase proteins
CABG: coronary artery bypass grafting
CAD: coronary artery disease
CAG: coronary angiography
CRP: C-reactive protein
FAI: fat attenuation index
FBG: fasting blood glucose
HR: heart rate
IL-6: interleukin-6
LDL-C: low-density lipoprotein cholesterol
MACE: major cardiovascular adverse events
MCP-1: monocyte chemotactic protein-1
NTLs: non-target lesions
OMT: optimal medical therapy
PCI: percutaneous coronary intervention
SAA-1: serum amyloid A-1
SBP: Systolic blood pressure
SI: stenosis index
TNF-α: tumor necrotic factor-α

## References

[1] Stone GW, Maehara A, Lansky AJ, de Bruyne B, Cristea E, Mintz GS (2011). A prospective natural-history study of coronary atherosclerosis. N Engl J Med.

[2] Erlinge D, Maehara A, Ben-Yehuda O, Bøtker HE, Maeng M, Kjøller-Hansen L (2021). Identification of vulnerable plaques and patients by intracoronary near-infrared spectroscopy and ultrasound (PROSPECT II): a prospective natural history study. Lancet.

[3] Seo YH, Kim YK, Song IG, Kim KH, Kwon TG, Bae JH (2019). Long-term clinical outcomes in patients with untreated non-culprit intermediate coronary lesion and evaluation of predictors by using virtual histology-intravascular ultrasound; a prospective cohort study. BMC Cardiovasc Disord.

[4] Wang J, Yan CY, Wang W, Wang TZ (2022). The clinical prediction factors for non-culprit lesion progression in patients with acute ST elevation myocardial infarction after primary percutaneous coronary intervention. BMC Cardiovasc Disord.

[5] Sekimoto T, Koba S, Mori H, Sakai R, Arai T, Yokota Y (2021). Small Dense Low-Density Lipoprotein Cholesterol: A Residual Risk for Rapid Progression of Non-Culprit Coronary Lesion in Patients with Acute Coronary Syndrome. J Atheroscler Thromb.

[6] Gaspardone A, Crea F, Versaci F, Tomai F, Pellegrino A, Chiariello L (1998). Predictive value of C-reactive protein after successful coronary-artery stenting in patients with stable angina. Am J Cardiol.

[7] Puri R, Nissen SE, Shao M, Ballantyne CM, Barter PJ, Chapman MJ (2013). Coronary atheroma volume and cardiovascular events during maximally intensive statin therapy. Eur Heart J.

[8] Räber L, Taniwaki M, Zaugg S, Kelbæk H, Roffi M, Holmvang L (2015). Effect of high-intensity statin therapy on atherosclerosis in non-infarct-related coronary arteries (IBIS-4): a serial intravascular ultrasonography study. Eur Heart J.

[9] Shah B, Pillinger M, Zhong H, Cronstein B, Xia Y, Lorin JD (2020). Effects of Acute Colchicine Administration Prior to Percutaneous Coronary Intervention: COLCHICINE-PCI Randomized Trial. Circ Cardiovasc Interv.

[10] Xu YL, Li JJ, Xu B, Zhu CG, Yang YJ, Chen JL (2011). Increased plasma C-reactive protein level predicts rapid progression of non-target atherosclerotic lesions in patients with stable angina after stenting. Chin Med J (Engl).

[11] Ma J, Liu X, Qiao L, Meng L, Xu X, Xue F (2021). Association Between Stent Implantation and Progression of Nontarget Lesions in a Rabbit Model of Atherosclerosis. Circ Cardiovasc Interv.

[12] Zuo L, Tian Z, Zhou B, Hou M, Chen Y, Han P (2024). Perivascular fat attenuation index value and plaque volume increased in non-target lesions of coronary arteries after stenting. Eur Radiol.

[13] Kashiyama K, Sonoda S, Muraoka Y, Suzuki Y, Kamezaki F, Tsuda Y (2015). Coronary plaque progression of non-culprit lesions after culprit percutaneous coronary intervention in patients with moderate to advanced chronic kidney disease: intravascular ultrasound and integrated backscatter intravascular ultrasound study. Int J Cardiovasc Imaging.

[14] Usui E, Matsumura M, Mintz GS, Zhou Z, Hada M, Yamaguchi M (2021). Clinical outcomes of low-intensity area without attenuation and cholesterol crystals in non-culprit lesions assessed by optical coherence tomography. Atherosclerosis.

[15] Waksman R, Di Mario C, Torguson R, Ali ZA, Singh V, Skinner WH (2019). Identification of patients and plaques vulnerable to future coronary events with near-infrared spectroscopy intravascular ultrasound imaging: a prospective, cohort study. Lancet.

[16] Lee TT, Feinberg L, Baim DS, Holmes DR, Aroesty JM, Carrozza JP Jr (2006). Effect of diabetes mellitus on five-year clinical outcomes after single-vessel coronary stenting (a pooled analysis of coronary stent clinical trials). Am J Cardiol.

[17] Al-Lamee R, Thompson D, Dehbi HM, Sen S, Tang K, Davies J (2018). Percutaneous coronary intervention in stable angina (ORBITA): a double-blind, randomised controlled trial. Lancet.

[18] Jo SH, Kim H, Kim HJ, Lee MH, Seo WW, Kim M (2024). Percutaneous coronary intervention versus medical therapy in stable angina: a matched cohort study. Heart.

[19] Hofma SH, Whelan DM, van Beusekom HM, Verdouw PD, van der Giessen WJ (1998). Increasing arterial wall injury after long-term implantation of two types of stents in a porcine coronary model. Eur Heart J.

[20] Tucker B, Vaidya K, Cochran BJ, Patel S (2021). Inflammation during Percutaneous Coronary Intervention-Prognostic Value, Mechanisms and Therapeutic Targets. Cells.

[21] Tenekecioglu E, Torii R, Katagiri Y, Chichareon P, Asano T, Miyazaki Y (2019). Post-implantation shear stress assessment: an emerging tool for differentiation of bioresorbable scaffolds. Int J Cardiovasc Imaging.

[22] Ding SF, Ni M, Liu XL, Qi LH, Zhang M, Liu CX (2010). A causal relationship between shear stress and atherosclerotic lesions in apolipoprotein E knockout mice assessed by ultrasound biomicroscopy. Am J Physiol Heart Circ Physiol.

[23] Gomes WJ, Buffolo E (2006). Coronary stenting and inflammation: implications for further surgical and medical treatment. Ann Thorac Surg.

