# Supplementary material for: Percutaneous coronary intervention as an independent predictor of non-target lesion progression in 1658 patients with coronary artery disease
Source: Theranostics. 2026 Jan 14;16(7):3857–69. doi: 10.7150/thno.125363 (PMC12846773; doi:10.7150/thno.125363)
Supplement: Supplementary file 1 — Supplementary tables and figure S1. [file thnov16p3857s1.pdf]

Supplemental materials

Table S1. Comparison of baseline characteristics between the final included cohort and eligible-but-excluded patients

Parameters	Included Cohort (n = 1658)	Eligible-but-excluded Patients (n = 2370)	*P* value
Age (years)	60.438±9.84	59.88±9.51	0.054
Sex (male, %)	1202(72.5%)	1654 (69.5%)	0.063
SBP (mmHg)	133(120，146）	131(121，145)	0.838
DBP (mmHg)	76(68, 84)	77(69, 84)	0.405
HR (bpm)	72(64, 80)	72(64, 80)	0.534
History of hypertension (n, %)	1073 (64.7%)	1533(64.7%)	0.983
History of diabetes (n, %)	515 (31.1%)	696 (29.4%)	0.248
History of smoking (n, %)	820 (49.5%)	1121 (47.3%)	0.177

The eligible-but-excluded patients (n = 2370) encompassed those with an inter-angiography interval outside 6-30 months (n = 2,041) and those within this time interval but meeting exclusion criteria (n = 329). The continuous variables were expressed as medians (Q1, Q3). DBP, diastolic blood pressure; HR, heart rate; bpm, beats per minute; SBP, systolic blood pressure.

Table S2. Changes in laboratory parameters of patients in NTLs progression and non-progression groups

Parameters	NTLs progression group (n = 1061)	NTLs non-progression group (n = 597)	*P* value
Δ Neutrophil ratio (%)	0.00(-5.65，6.30)	-0.30(-6.10，5.50)	0.137
ΔTC (mmol/L)	-0.52(-1.14，0.03)	-0.47(-1.20，0.07)	0.905
ΔLDL-C(mmol/L)	-0.43(-0.92，-0.01)	-0.42(-0.96，0.04)	0.674
ΔHDL-C(mmol/L)	-0.02(-0.17，0.11)	-0.01(-0.14，0.10)	0.583
ΔTG (mmol/L)	-0.14(-0.49，-0.17)	-0.12(-0.46，0.18)	0.536
ΔCreatinine (μmol/L)	1.00(-5.00，8.00)	1.00(-5.00，7.00)	0.171
ΔFBG (mmol/L)	-0.04(-0.64，0.47)	-0.02(-0.66，0.76)	0.052
ΔUric acid (μmol/L)	-8.00(-46.50，37.50)	-5.00(-30.00，22.00)	0.642
ΔHomocysteine (μmol/L)	-0.45(-3.07，2.10)	-0.50(-3.10，2.00)	0.669

The continuous variables were expressed as medians (Q1, Q3). The changes (Δ) of each laboratory parameter were calculated by the formula: Δ value = value on the second admission — value on the first admission. FBG, fasting blood glucose; HDL-C, high-density lipoprotein cholesterol; LDL-C, low-density lipoprotein cholesterol; NTLs, non-target lesions; TC, total cholesterol; TG, triglycerides.

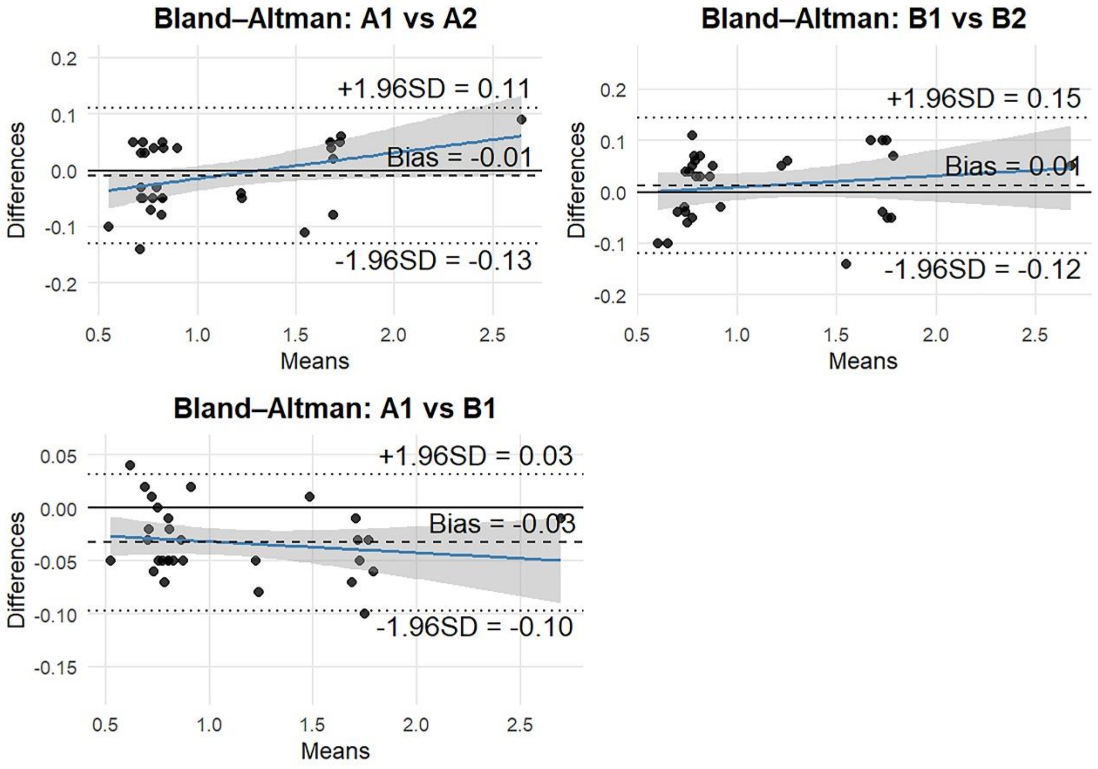

Figure S1. Bland-Altman analysis of intra- and inter-observer differences for stenosis index of non-target lesions.

Thirty randomly selected angiograms were analyzed by two observers blinded to clinical data and in randomized order. A. Bland-Altman plot for intra-observer variability of the first observer (Observer 1). B. Bland-Altman plot for intra-observer variability of the second observer (Observer 2). C. Bland-Altman plot for inter-observer variability between the two observers. Intraclass correlation coefficient (ICC) analysis demonstrated excellent reproducibility: for inter-observer ICC = 0.983, and for intra-observer ICCs = 0.985 and 0.975 for the two observers, respectively. SI, stenosis index; NTLs, non-target lesions.
